# The bladder microbiome of NMIBC and MIBC patients revealed by 2bRAD-M

**DOI:** 10.3389/fcimb.2023.1182322

**Published:** 2023-06-07

**Authors:** Jian-Xuan Sun, Qi-Dong Xia, Xing-Yu Zhong, Zheng Liu, Shao-Gang Wang

**Affiliations:** Department and Institute of Urology, Tongji Hospital, Tongji Medical College, Huazhong University of Science and Technology, Wuhan, China

**Keywords:** bladder cancer, microbiome, 2bRAD-M, MIBC, NMIBC

## Abstract

**Background:**

Bladder cancer (BCa) is the most common malignancy of the urinary tract which can be divided into non-muscle-invasive bladder cancer (NMIBC) and muscle-invasive bladder cancer (MIBC), and their microbial differences are not fully understood. This study was conducted by performing 2bRAD sequencing for Microbiome (2bRAD-M) on NMIBC and MIBC tissue samples to investigate the microbiota differences between NMIBC and MIBC individuals.

**Methods:**

A total of 22 patients with BCa, including 7 NMIBC and 15 MIBC, were recruited. Tumor tissues were surgically removed as samples and DNA was extracted. Type IIB restriction endonucleases were used to enzymatically cleave the microbial genome for each microbe’s tag and map it to species-specific 2bRAD markers to enable qualitative and quantitative studies of microbes between MIBC and NMIBC tissues.

**Results:**

A total of 527 species were detected. The microbial diversity of NMIBC tissues was significantly higher than that of MIBC tissues. Microbial composition of the two tumor tissues was similar, where Ralstonia_sp000620465 was the most dominant species. 4 species (Acinetobacter_guillouiae, Anoxybacillus_A_rupiensis, Brevibacillus_agri and Staphylococcus_lugdunensis) were enriched in NMIBC, while Ralstonia_mannitolilytica, Ralstonia_pickettii, and Ralstonia_sp000620465 were overrepresented in MIBC. 252 discriminatory character taxa were also revealed by linear discriminant analysis effect sizea (LEfSe). Species importance point plots identified Ralstonia_sp000620465, Cutibacterium_acnes and Ralstonia_pickettii as the three most important species between the two groups. Meanwhile, functional annotation analysis showed 3011 different COGs and 344 related signaling pathways between MIBC and NMIBC microbiome.

**Conclusion:**

This first 2bRAD-M microbiome study on MIBC and NMIBC tissues revealed significant differences in the microbial environment between the two groups, which implies a potential association between tumor microbial dysbiosis and BCa, and provides a possible target and basis for subsequent studies on the mechanisms of BCa development and progression.

## 1 Introduction

Bladder cancer (BCa) is the most common malignancy of the urinary tract and is the 10th most common cancer worldwide (Babjuk et al., 2022; Lobo et al., 2022). Depending on the depth of invasion of the tumor into the bladder wall, BCa can be divided into two categories: non-muscle-invasive bladder cancer (NMIBC) and muscle-invasive bladder cancer (MIBC). In NMIBC, tumors are confined to the mucosa and submucosa and include carcinoma in situ, pTa and pT1 tumors, according to the pathologic TNM (pTNM) staging system, whereas in MIBC, tumor cells invade the muscularis and include pT2-pT4 tumor stages (Lenis et al., 2020). For NMIBC, transurethral resection of the bladder tumor (TURBT) and postoperative chemotherapy are the main treatment options, while for MIBC, radical cystectomy (RC) plus pelvic lymph node dissection (PLND) are the gold standard of treatment (Tran et al., 2021; Witjes et al., 2021; Babjuk et al., 2022). With the progress of research, some novel and effective diagnostic and therapeutic techniques for BCa have appeared (Chen et al., 2020; Ruan et al., 2021; Tran et al., 2021), but the high recurrence of non-invasive BCa and the high mortality of invasive BCa remain urgent issues (Dobruch and Oszczudłowski, 2021). Therefore, a more comprehensive understanding of BCa microenvironment and development is needed to better predict disease and/or provide a more informative basis for treatment.

Factors currently considered to predispose BCa include smoking (Freedman et al., 2011; van Osch et al., 2016), age, gender, family history, genetic factors, occupational exposure to carcinogenic chemicals (Pashos et al., 2002), radiological factors, metabolic disorders and schistosomiasis infection and chronic urinary tract inflammation (Teleka et al., 2018; Yu et al., 2018). Meanwhile, microbial environmental status has been recently taken into consideration as a causative factor for BCa. The microbiota has been found to be associated with a variety of cancers (Garrett, 2015) and, in particular, the gut microbiome exerts significant roles in local and distant tumorigenesis (Li et al., 2019; Si et al., 2021). Due to the limitations of detection techniques, some thought that the bladder was a sterile environment. With the development of PCR technology and 16S rRNA sequencing, the knowledge of microbes colonizing the bladder and other urinary tracts were expanded (Whiteside et al., 2015). Before long, several studies identified that these microbes were associated with multiple urologic diseases ranging from urinary incontinence to interstitial cystitis (Siddiqui et al., 2012; Thomas-White et al., 2017). Nevertheless, the relationship between the microbiota and bladder cancer is still unclear. Several recent studies have found that the urobiome of BCa patients may differ from that of healthy individuals, but no consistent conclusions have been drawn (Wu et al., 2018; Chipollini et al., 2020; Hussein et al., 2021; Oresta et al., 2021). Notably, most of the studies were performed on patients’ urine, which is not fully representative of the intravesical pattern and can pose a risk of sample staining (Mansour et al., 2020). Meanwhile, the limitations of techniques such as 16S rRNA that cannot be balanced with cost and precision to the species level may be another reason for the inconsistent results.

Current studies on the urinary tract microbiota have mainly used 16S rRNA sequencing and, most recently, metagenomic sequencing. Since this technique sequences only the 16S rRNA genes of microbes, it can generally provide classification to the genus level, but is difficult to provide precise determination at the species level. Although whole macrogenome sequencing technology permits the sequencing of whole genomes within a sample, which enables microbiota differentiation at the species level, it is costly and difficult to carry out in large sample populations. Meanwhile, this technique often requires a high volume of detectable DNA, but it is difficult to obtain as much biomass from the urinary microbiota as from the gut microbiota. In order to achieve low-cost and high-precision bladder microbiota analysis, alternative assays need to be sought. Here, we used 2bRAD microbiome sequencing (2bRAD-M), a sequencing method to investigate the microbiome (Sun et al., 2022). This method uses type IIB restriction endonucleases to enzymatically cleave the microbial genome for each microbe’s tag and map it to species-specific 2bRAD markers to enable qualitative and quantitative studies of microbes (Wang et al., 2012; Wang et al., 2016). In this way, 2bRAD-M enables accurate and cost-effective analysis of low biomass microbiota at the species level (Sun et al., 2022).

The current study was conducted by performing 2bRAD-M on 22 groups of tissue samples including NMIBC and MIBC to determine whether the microbiota differs between NMIBC and MIBC individuals. To our knowledge, this is the first study to investigate the bladder microbiome of BCa patients at the species level with 2bRAD-M.

## 2 Methods

### 2.1 Recruitment of patients

**Figure 1:**
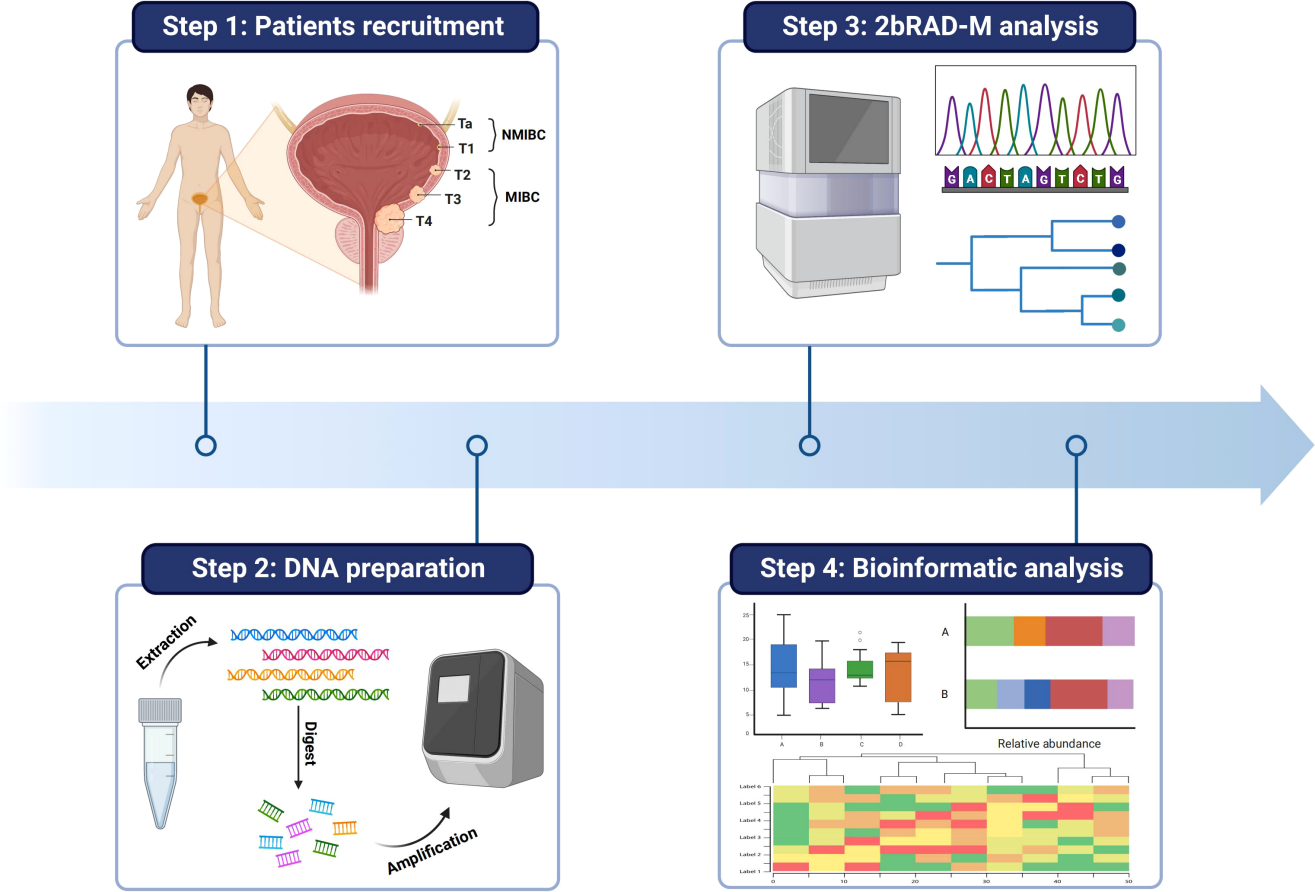
Schematic diagram of the study flow. Tumor samples from MINC and NMIBC patients were first collected. Then the DNA information of microbes in the samples was extracted and the microbes were compared and analyzed at the species level by 2bRAD-M technology. Finally, the data information was analyzed by multidimensional processing.

As shown in **Figure 1**, we first recruited MIBC and NMIBC patients who were admitted to Tongji Hospital (Wuhan, China). In order to minimize confounding factors that could affect the microbiota of the bladder tissue, strict exclusion criteria were established: urinary tract infections (UTIs), urinary tract disease other than BCa, and antibiotic treatment or catheterization within 4 weeks. All patients underwent preoperative imaging evaluation as well as surgical resection by the same treatment team. Tumor tissue removed during surgery was collected for microbiological component analysis, and clinical information was collected for these patients.

### 2.2 Sample collection and processing

The tumor tissues were stored at -80°C immediately after surgery, and all procedures were performed under aseptic conditions. BCa tissue collection was approved by Ethical Review Board of Tongji Hospital, Tongji Medical College, Huazhong University of Science and Technology (2021S055), and informed consents were obtained from patients for the use of samples.

### 2.3 DNA extraction, library preparation and sequencing

The genomic DNA were extracted by TIANamp Micro DNA Kit (Tiangen, cat.#DP316). The 2bRAD-M library was prepared following the protocol of Wang et al (Wang et al., 2012) with slight modifications, where the endonuclease enzyme was changed from BsaXI enzyme to BcgI enzyme. DNA (1 pg-200 ng) was digested with 4 U of BcgI enzyme (NEB, cat. no. R0545) for 3 h at 37°C. The aptamer was then ligated to the DNA fragment. The ligation reaction was performed by mixing 5 µl of digested DNA with 10 µl of ligation master mix containing 0.2 µm each of two aptamers and 800 U of T4 DNA ligase (NEB), and was performed at 4°C for 12 hours. The ligation product was then amplified by PCR and placed on an 8% polyacrylamide gel at 400 V for 35 min. Bands of approximately 100 bp were excised from the polyacrylamide gel and the DNA was diffused from the gel into nuclease-free water for 12 hours at 4°C. PCR was performed with platform-specific barcode primers to introduce sample-specific barcodes. Each 20 µl PCR contains 25 ng of gel-extracted PCR product, 0.2 µM of each primer, 0.3 mM dNTP, 1× Phusion HF buffer and 0.4 U Phusion high-fidelity DNA polymerase (NEB). PCR products are purified using the QIAquick PCR Purification Kit (Qiagen, cat.#28104), followed by Illumina Nova PE150 platform for sequencing. 2bRAD-M was performed at OE BioTech Co., Ltd., Qingdao. Details of the adapters and primer sequences are listed in **Supplementary Table 1**.

### 2.4 Sequencing processing and quantitative analysis

Based on the recognition site of type IIB restriction endonuclease, reads were scanned and sequences containing enzyme slices were extracted, and clean reads were generated according to the following conditions (1): removing reads containing more than 8% of unknown bases (2); removing low quality reads (the number of bases with quality value lower than Q30 exceeds 20% of the bases of the whole reads). Classification was performed using the unique 2bRAD tag database containing bcgI-derived tags of taxonomic species from 173,165 microbial genomes (comprising bacteria, bacteriophages and archaea). First, the quality-controlled 2bRAD markers were mapped to the 2bRAD marker database using the built-in Perl script. To control for false positives in species identification, a G score was assigned to each species in the sample: G score _species i_ = $\sqrt{s_{i}\times t_{i}}$ (S: the number of reads assigned to all 2bRAD tags belonging to species i in the sample. t: the number of all 2bRAD tags of species i having been sequenced in the sample). The G value is the harmonious average of the read coverage of 2bRAD markers belonging to a species and the number of all possible 2bRAD markers for this species, and here the threshold for G value to find false positives was set to 5 (Sun et al., 2022). Next, the average read coverage of all 2bRAD markers for each species is calculated, which represents the number of individuals belonging to a species in a sample at a given sequencing depth. Finally, the relative abundance of a particular species is calculated according to the formula: Relative abundance _species i_ = $\frac{s_{i}/T\text{i}}{\sum_{i=1}^{n}s_{i}/Ti}$ .

### 2.5 Bioinformatic analysis

We then calculated the alpha diversity including Chao1 (estimating the actual species number in the community by counting low abundance ones), Shannon (reflecting species richness and evenness of distribution) and Simpson (reflecting community diversity level) index with “vegan” package and visualize it as box plots (Oksanen et al., 2015). For beta diversity, including Bray-Curtis (characterizing differences in species abundance), Binary Jaccard (comparing the probability of similarity and dispersion in a sample set) and Euclidean distances (reflecting the actual distance between the abundance of two species groups in the multidimensional space) were estimated by the “ Vegan “ package and displayed as Principal Coordinate Analysis (PCoA) scatter plots. Linear discriminant analysis (LDA) effect sizea (LEfSe) was used to identify differential taxa between groups, with an LDA score threshold of 4.0 (Segata et al., 2011). Clusters of Orthologous Groups (COG) and Kyoto Encyclopedia of Genes and Genomes (KEGG) analysis were applied to predict functions of the urobiome.

### 2.6 Statistical analysis

Statistical analysis was performed using SPSS (version 26) and R software (version 4.1.1). Group comparisons for alpha diversity were assessed by Wilcoxon test and for beta diversity by permutational multivariate analysis of variance (PERMANOVA). Kruskal Wallis analysis was used to compare the differences in microbial communities between MIBC and NMIBC. For functional prediction (COG and KEGG), differences between groups were analyzed by Wilcoxon test. P-value< 0.05 was considered statistically significant.

## 3 Results

### 3.1 Clinical characteristics of the included patients

A total of 22 patients were enrolled in this study, 7 patients with NMIBC and 15 patients with MIBC. The enrolled patients were predominantly male, with an average age of over 60 years. All MIBC patients underwent RC, while NMIBC patients underwent mainly electrosurgery.

### 3.2 Diversity of BCa tissue microbiota

**Figure 2:**
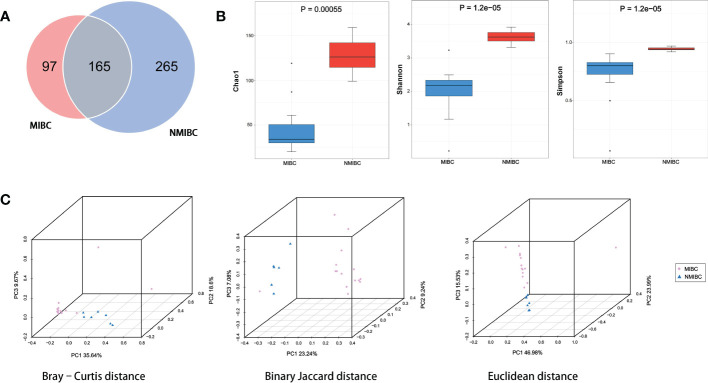
Variation in tumor microbial diversity and structure between the MIBC and NMIBC groups. **(A)** The Venn diagram shows the number of species contained in the MIBC and NMIBC groups and the relationship between them. **(B)** Comparison of alpha diversity (Chao1, Shannon and Simpson) between the two groups. **(C)** Comparison of beta diversity (Bray-Curtis distance, Binary Jaccard distance and Euclidean distance) between the two groups. 3D-PCoA plots show the results of the analysis based on the three distance matrices for the differences between the two groups, each point in the plot represents a sample, the same color for the same grouping.

The details of the quality control during the assay are described in **Supplementary Table 2**. A total of approximately 159 million clean reads were obtained from 22 bladder tissue samples, with an average of approximately 7.24 million reads per sample. These reads obtained were compared with 2b-Tag-DB and categorized into 527 microbial categories at the species level. As shown in the Venn diagram (**Figure 2A**), 165 microbial species were shared between NMIBC and MIBC. 265 species are exclusively in the NMIBC group, and another 97 species are covered by the MIBC group. Then alpha diversity analysis was performed. Chao1 was calculated to assess the community richness of bladder tissues, and the Shannon and Simpson indices were evaluated to assess community diversity. Chao1, Shannon’s index and Simpson’s index were statistically significantly different among the two groups (**Figure 2B**). Specifically, the NMIBC group was significantly superior to the MIBC group in terms of both richness and diversity. Similar results can be seen in the beta diversity analysis (**Figure 2C**). The microbiota of the NMIBC group showed differences from the MIBC group in terms of Bray-Curtis distance (P< 0.001), Binary Jaccard distance (P< 0.001) and Euclidean distance (P< 0.01).

### 3.3 Bacterial community composition in the BCa microbiota

**Figure 3:**
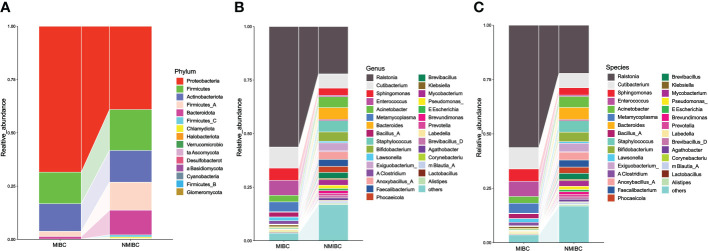
The composition and relative abundance of microorganisms in the MIBC and NMIBC groups. **(A)** Relative abundance at the phylum level. **(B)** Top 30 most abundant genera. **(C)** Top 30 most abundant species.

11 and 15 phylum were identified in MIBC and NMIBC tissues, respectively (**Figure 3**). The predominant phylum included Proteobacteria (68.45 and 39.09%), Firmicutes (14.76 and 19.17%) and Actinobacteriota (12.98 and 14.92%), while Firmicutes_A (13.13%) and Bacteroidota (11.55%) also showed high abundance in the microbial phylum of NIMBC. There were 147 genera found in MIBC tissues and another 222 were found in that of NMIBC. Ralstonia (56.29 and 22.16%) and Cutibacterium (9.82 and 6.60%) were the dominant genera common to both groups. Moreover, Enterococcus (6.91%), Sphingomonas (5.77%) and Metamycoplasma (4.60%) were also the dominant genera in MIBC tissues, while for NMIBC the dominant genera also included Bacteroides (5.51%) and Staphylococcus (5.27%) and Acinetobacter (5.07%), among others. When considered at the species level, Ralstonia_sp000620465 was most common in both groups of species, with other common species including Ralstonia_pickettii and Cutibacterium_acnes. The former three together with Enterococcus_faecalis and Sphingomonas_paucimobilis as well as Anoxybacillus_A_rupiensis and Staphylococcus_lugdunensis constitute the five common species in MIBC and NMIBC, respectively.

### 3.4 Differential abundance of microbial taxa between MIBC and NMIBC tissues

**Figure 4:**
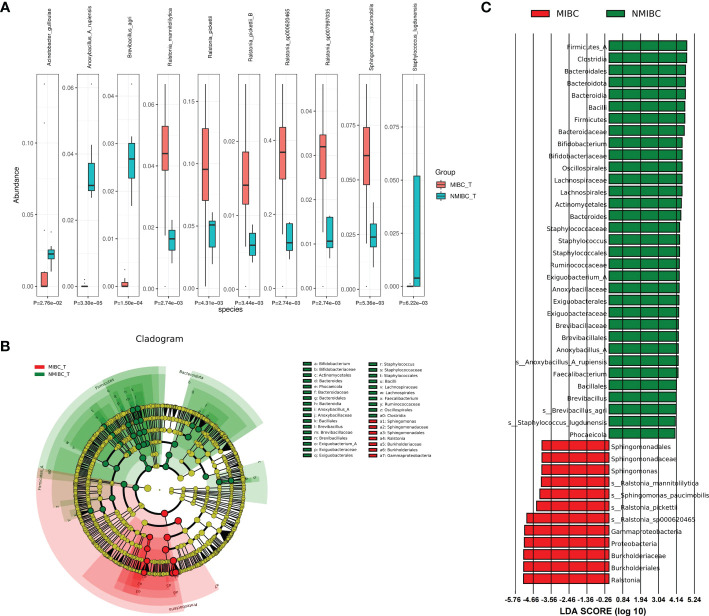
Comparison of differences in abundance of microbial taxa between MIBC and NMIBC groups. **(A)** The top 10 species with significant differences. **(B)** Differential species annotated branching plots represent the taxonomic hierarchy of the discriminatory biomarkers identified with LEfSe. **(C)** Differential species score plots present the species with relatively high abundance in each of the two groups.

To characterize the differential representation of microorganisms between MIBC and NMIBC tissues, we performed Kruskal Wallis statistical analysis of taxa differences between the two groups (**Table 1**; **Figure 4A**; **Supplementary Figure 1**). At the Phylum level, the abundance of Proteobacteria increased significantly within the MIBC tissue, and in contrast, the MIBC group had significantly lower abundance of Bacteroidota, Firmicutes and Firmicutes_A than the NMIBC group. For Genus, the abundance of Ralstonia and Sphingomonas was significantly higher in MIBC tissues, while Anoxybacillus_A and others showed higher abundance in NMIBC tissues. At the species level, Ralstonia_mannitolilytica, Ralstonia_pickettii, Ralstonia_pickettii_B, Ralstonia_sp000620465, Ralstonia_sp007997035 and Sphingomonas_paucimobilis were the species significantly more enriched in MIBC, whereas Acinetobacter_guillouiae, Anoxybacillus_A_rupiensis, Brevibacillus_agri and Staphylococcus_lugdunensis showed more enriched in the MIBC group (**Figure 4A**).

**Table 1:** Comparison of the mean relative abundance of tumor microbiota in MIBC and NMIBC groups at the phylum, genus and species levels, respectively.

Taxa		Average relative abundance (%)		
		MIBC	NMIBC	P value
Phylum	Bacteroidota	0.011673	0.115487	0.000399
	Firmicutes	0.147612	0.191681	0.015019
	Firmicutes_A	0.024492	0.131292	0.002144
	Firmicutes_C	0.000332	0.009899	3.63E-05
	Proteobacteria	0.684522	0.390905	0.005363
	Verrucomicrobiota	0	0.002248	0.007881
Genus	Anoxybacillus_A	0.000203	0.036286	3.30E-05
	Bacteroides	0.002265	0.055104	3.63E-05
	Bifidobacterium	0.000645	0.043677	0.001945
	Brevibacillus	0.000596	0.027646	0.00015
	Exiguobacterium_A	0	0.037773	0.034098
	Faecalibacterium	0.000837	0.03257	9.05E-05
	Phocaeicola	0.002254	0.028082	0.004294
	Ralstonia	0.562908	0.221618	0.002737
	Sphingomonas	0.057656	0.033994	0.021966
	Staphylococcus	7.92E-05	0.052745	0.001045
Species	Acinetobacter_guillouiae	0.021608	0.022858	0.027606
	Anoxybacillus_A_rupiensis	0.000203	0.035788	3.30E-05
	Brevibacillus_agri	0.000596	0.027397	0.00015
	Ralstonia_mannitolilytica	0.043369	0.015761	0.002737
	Ralstonia_pickettii	0.096869	0.043631	0.004305
	Ralstonia_pickettii_B	0.01509	0.006095	0.003441
	Ralstonia_sp000620465	0.348925	0.133581	0.002737
	Ralstonia_sp007997035	0.029419	0.012022	0.002737
	Sphingomonas_paucimobilis	0.057519	0.024614	0.005363
	Staphylococcus_lugdunensis	7.92E-05	0.028992	0.006223

Meanwhile, we employed LEfSe analysis here to reveal the composition of differential taxa between the two biome groups (**Figures 4B, C**). The results of LEfSe analysis revealed 252 discriminatory character taxa, which differed significantly in abundance between the MIBC and NMIBC groups. At the genus level, the abundance of Sphingomonas was significantly higher in the MIBC tissues, while Bifidobacterium, Staphylococcus, Exiguobacterium_A, Anoxybacillus_A and Faecalibacterium were the differential genera that tended to be enriched in the NMIBC group. At the species level, those enriched in NMIBC included Ralstonia_sp000620465, Ralstonia_pickettii, Sphingomonas_paucimobilis and Ralstonia_mannitolilytica, and those with higher abundance in NMIBC included Anoxybacillus_A_rupiensis, Brevibacillus_agri and Staphylococcus_lugdunensis.

### 3.5 Model prediction analysis between MIBC and NMIBC tissues

**Figure 5:**
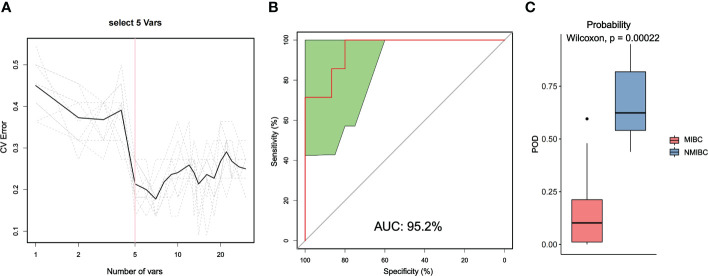
Distinction between MIBC and NMIBC groups by random forest analysis. **(A)** Cross-validation error curves showed that five species markers were selected as the best set of markers. **(B)** The mean AUC between the two groups reached 95.2%. **(C)** The POD values were significantly higher in the NMIBC group than in the MIBC group.

We also used random forest analysis to depict species importance among the top 30 species in relative abundance (**Figure 5**; **Supplementary Figure 2**). Species importance point plots showed that Ralstonia_sp000620465, Cutibacterium_acnes and Ralstonia_pickettii were the three most important species between the MIBC and NMIBC groups.

Then, we constructed a random forest classifier and selected five species as the best marker set to distinguish MIBC and NMIBC (**Supplementary Table 3**). The performance of this optimal marker set was examined by ROC analysis, which showed a mean AUC value of 95.2% between the MIBC and NMIBC groups. The probability of disease (POD) values refers to the ratio of the number of random-generated decision trees that predict samples of NMIBC to that of MIBC. Obviously, the POD were significantly higher in the NMIBC group than that in the MIBC group (P=0.00022), which indicates that the POD based on microbial species markers has a strong diagnostic potential to identify NMIBC from MIBC.

### 3.6 Differences in predictive functions between MIBC and NMIBC

**Figure 6:**
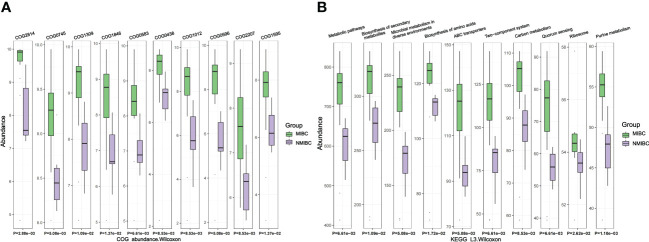
Differences in microbial function between groups by functional annotation analysis. **(A)** Results of COG function prediction for the top 10 most significant differences. **(B)** Results of KEGG function prediction for the top 10 most significant differences.

For the identified microbiota, we further predicted their functions and used the Wilcoxon test for comparison between the two groups. We identified 3011 COGs that differed between MIBC and NMIBC, with the top five COGs being COG2814 (Predicted arabinose efflux permease AraJ, MFS family), COG0745 (DNA-binding response regulator, OmpR family, contains REC and winged-helix (wHTH) domain), COG1309 (DNA-binding protein, AcrR family, includes nucleoid occlusion protein SlmA), COG1846 (DNA-binding transcriptional regulator, MarR family) and COG0583 (DNA-binding transcriptional regulator, LysR family) (**Figure 6A**). Analysis based on the KEGG database revealed 344 related signaling pathways between MIBC and NMIBC groups, and the top five pathways with significant differences included metabolic pathways, biosynthesis of secondary metabolites, microbial metabolism in diverse environments, biosynthesis of amino acids and ABC transporters (**Figure 6B**).

## 4 Discussion

BCa is a complex urothelial tract tumor whose etiology is complex and often involves a combination of genetic and environmental factors. In the initial stages of research, the healthy urinary tract was considered sterile due to the limitations of the microbial culture methods used. With the help of 16S rRNA sequencing technology, a symbiotic microbial system was identified in the bladder (Lewis et al., 2013; Whiteside et al., 2015). This discovery has led to the identification of microbial associations in a variety of diseases (Whiteside et al., 2015). At the same time, several studies have identified the microbiota as an integral part of tumor development, making the causal association between the bladder microbiota and BCa a concern. Although there is insufficient evidence for the involvement of bladder microbiota in any of the processes of BCa, the widespread use of BCG (Han et al., 2020), an attenuated tuberculosis vaccine, in the treatment of NMIBC suggests that microorganisms can be involved in BCa processes. At the same time, chronic irritation of the urinary epithelium has been shown to be a possible trigger of BCa (Pelucchi et al., 2006; Yu et al., 2018), which provides a good hypothesis for the involvement of microbiota in BCa development. In recent years, differences in the composition or abundance of microbiota observed in BCa patients and healthy populations have further implied its potential importance in the pathogenesis of BCa (Chipollini et al., 2020; Hussein et al., 2021; Oresta et al., 2021). Unfortunately, the 16S rRNA sequencing methods currently used for bladder microbiota studies have difficulties in achieving the accuracy of detection down to the species level, which limits the understanding of the bladder microbiota. Also, the source of samples for most studies is the urine of BCa patients, which has the problem of sample contamination. Even though the transurethral catheter was used to collect the clean urine, the microbes in the urine are still not a complete substitute for the real situation in the bladder. Here, we aimed to analyze the microbiota of tissue samples from bladder cancer patients using 2bRAD-M technology, a new microbial sequencing technique, in order to accurately characterize the microbial landscape within the cancerous bladder at the species level.

Both alpha and beta diversity analyses showed a significant lower microbial diversity within MIBC tumor tissue. Comparative data on the microbiota diversity of MIBC and NMIBC tissues are lacking. Fei Liu et al. (2019) compared BCa tumor tissue with tumor-free tissue and found that the Shannon diversity of tumor tissue was significantly lower than that of tumor-free tissue, but they did not classify the tumor period. Therefore, a larger sample size and careful study may be helpful to clarify the difference in microbial diversity between tumor and tumor-free tissues. Based on our results and combined with the association between reduced gut microbial diversity and colon cancer development (Ahn et al., 2013), perhaps lower microbial diversity represents more severe BCa.

The results of the microbiota composition analysis showed similarities in taxa composition between MIBC and NMIBC tissues. At the phylum level, the two groups share a similar composition of dominant microorganisms (Proteobacteria, Firmicutes and Actinobacteriota), and the results of the urinary microorganism study by Hai Bi et al. (2019) indicate that they are all conserved constituents of the urine. Similarly, Ralstonia and Cutibacterium are the dominant genera shared by both groups, but differ in the composition of the remaining genera. Enterococcus is the third most abundant microbial genus in MIBC tissues, but some studies have shown that it is also enriched in the urine of healthy men (Fouts et al., 2012). Bacteroides, Staphylococcus and Acinetobacter were enriched in NMIBC. Previous studies have reported a high prevalence of Bacteroides in BCa tissue (Mansour et al., 2020), which is not limited to NMIBC but can also be observed in MIBC tissue (Chipollini et al., 2020). Notably, enrichment of Staphylococcus was also observed in the study by Jiarong Zeng et al. (2020), but evidences also suggested its enrichment in normal urine (Pearce et al., 2014; Curtiss et al., 2017). The accumulation of Acinetobacter in BCa has also been widely reported (Wu et al., 2018; Liu et al., 2019), and a relationship with increased bacterial diversity has been proposed (Wu et al., 2018). It is worth noting that Acinetobacter is an important genus causing drug resistance and multiple diseases (Dijkshoorn et al., 2007; Ketter et al., 2018), and its increased content with Proteobacteria may be a predictor of BCa occurrence (Friedrich and Choi, 2022). Our results suggest a greater predominance of Acinetobacter in NMIBC, which may be a contributing factor to the greater microbial diversity in the NMIBC group. At the species level, Ralstonia_sp000620465, Ralstonia_pickettii and Cutibacterium_acnes were the dominant species shared by the MIBC and NMIBC groups. The first two are consistent with the dominance of Ralstonia at the genus level, and they are both microorganisms present in the environment that may be associated with the occurrence of cystic fibrosis (Stelzmueller et al., 2006; Prior et al., 2017).Cutibacterium_acnes is a cutaneous commensal that plays an essential role in the mechanism of acneogenesis (Platsidaki and Dessinioti, 2018).

Kruskal Wallis and LEfSe analyses provided more information on the differences in microbial richness levels between groups. the abundance of Proteobacteria was higher in MIBC tissues than in NMIBC, while Bacteroidota and Firmicutes showed the opposite results. It was found that NMIBC patients enriched in Firmicutes phylum microorganisms such as Lactobacillus were less likely to experience recurrence of BCG treatment (Sweis et al., 2019). Therefore, Firmicutes may have therapeutic or survival predictive value in NMIBC patients. At the genus level, the richness of Ralstonia and Sphingomonas was significantly higher in MIBC tissues, while Bifidobacterium et al. showed higher abundance in NMIBC tissues. In 2019, Liu et al. (2019) identified high expression of Ralstonia in BCa tissues, and meanwhile, the presence of Ralstonia in BCa was similarly highlighted in a recent study by Bukavina et al. (2023) Sphingomonas is widely present in the environment and has an environmental restorative role (Asaf et al., 2020). Hyun Kyu Ahn et al. (2022) revealed that, compared with other urological tumors, there was a higher content of Sphingomonas in BCa compared to other urological tumors. At the same time, Sphingomonas was found to be more abundant in tumor tissues than in urine samples, suggesting its potential role as a participant in the process of BCa development (Mansour et al., 2020). Hence, a more enriched Sphingomonas may suggest a more severe BCa condition (i.e. MIBC). Bifidobacterium has an important role in the microbial homeostasis and anti-inflammatory response of mucosa (Di Giacinto et al., 2005; Turroni et al., 2011), and can inhibit IL-6 secretion (Trikha et al., 2003). Microbial homeostasis imbalance and inflammatory response are important mechanisms of tumorigenesis, and these evidences indicate that Bifidobacterium may be one of the factors affecting tumors. The study by Vyara Matson et al (Trikha et al., 2003). revealed a relationship between enriched Bifidobacterium and better response to immunotherapy in melanoma patients. In our study, the abundance of Bifidobacterium was lower in MIBC than in NMIBC, suggesting that it may be an indicator of bladder mucosal damage and more likely a protective factor for BCa.

Random forest analysis screened Ralstonia_sp000620465, Cutibacterium_acnes and Ralstonia_pickettii as three important species for the intergroup classification of MIBC and NMIBC, all of which tended to be enriched in MIBC. A selected optimal microbial set containing 5 species showed strong potential to differentiate MIBC from NMIBC tumors with an average AUC value of 95.2%. The microbes in this set of clusters may be potential markers for different BCa types and implicate their association with BCa progression.

We also predicted COG annotation and KEGG pathways and compared them between the two groups. Our results indicate that there is a wide range of significant differences in microbiome function between MIBC and NMIBC tissues. This may hint at the shaping of microbiome function of bladder tissue by the microbiota, which could further alter the status of BC by affecting its relationship with the host. The results of KEGG analysis revealed that microbes in MIBC tissues were more active in metabolic pathways. In particular, the ABC transporter serves as an important tool for substrate transport (Liston et al., 2017) and the two-component system is a sophisticated pathway for regulating signal transduction and gene expression (Chang and Stewart, 1998), and their increased enrichment in MIBC microorganisms may be responsible for the active metabolism. A more detailed mechanistic study will be necessary to better understand the processes involved.

Based on the available microbiome data of BCa tissues, the reasons for BCa progression may not be caused by a single microbial alteration. A large number of microorganisms are present in the bladder, and the bladder mucosa is an excellent vehicle for microorganisms to host or adhere to. The degree of tumor invasion then tends to alter the microorganisms and their environment of survival. In turn, an increase or decrease in microbial species or an alteration in abundance may reduce protection and affect the inflammatory response, thus contributing to tumor progression. Here, we hypothesized that imbalance in bladder microbial homeostasis is a potential factor contributing to BCa progression.

There are several limitations of our study. First, as for the samples, we categorized the BCa samples according to tumor stage (NMIBC and MIBC), but each category contained a limited sample size. Meanwhile, we took quality control measures during the sample collection and detection stages, but contamination is not 100% avoidable, and a larger sampling size analysis as conditions permit would make the conclusions more representative and generalizable. Also, the surgical procedures were selected based on the principle of optimal prognosis for the patients. We avoided surgical margins in the sample selection process to minimize the impact of the surgery itself on the microbiological environment, but the between-group variation due to different surgical approaches may still be an issue that needs attention. Second, in terms of detection methods, the 2bRAD-M technology has restrictions in predicting potential functional pathways in the microbiome, which is a weakness compared to macrogenome sequencing technologies. Also incomplete reference databases and limited resolution for strain-level identification may lead to deviations of the assay results from the actual ones, although we tried to avoid this as much as possible in the assay process. Finally, as for the type of our study, it is limited to data analysis based on the observation of bladder microbiome characteristics, and despite its significance, it is not yet possible to provide an accurate description of the its logical relationship with BCa. Basic mechanistic studies as well as clinical follow-up studies may be necessary in order to investigate more specifically in what form the bladder microbiota is involved in the development and progression of bladder cancer.

The relationship between microbes and tumors has been the topical issue for research, and 11 microorganisms have been clearly classified as carcinogens (Biological Agents, 2012). Monitoring and elimination measures against these microbes are often important initiatives for cancer prevention. In the therapeutic field of oncology, on one hand, microbes such as BCG and oncolytic viruses have become important therapeutic interventions for tumors such as NMIBC and melanoma (Lenis et al., 2020; Mondal et al., 2020); and on the other hand, the microbial environment of the tumor may represent an important factor influencing treatment. Although there is a lack of reports in BCa, the commensal microbiology of the organism has been shown to be a potential factor influencing the response of metastatic melanoma to anti-PD-1 therapy (Matson et al., 2018). Therefore, our analyses on the MIBC and NMIBC microbiota may provide novel approaches in BCa treatment. The features of the bladder’s microbial environment and its balance in BCa therapies may attract more attention in the future.

## 5 Conclusion

In conclusion, our study is the first to characterize differences in the microbial environments of MIBC and NMIBC tissues with 2bRAD-M, a novel technique that enables taxonomic analysis of low biomass samples at the species level. Altogether, the microbial diversity of NMIBC tissues was higher than that of MIBC. Both tissues had similar microbial composition, but elevated levels of Ralstonia_sp000620465, Cutibacterium_acnes and Ralstonia_pickettii were observed in MIBC tissues, while Acinetobacter_guillouiae, Anoxybacillus _A_rupiensis and Brevibacillus_agri were more enriched in NMIBC tissues. We speculate that imbalance of bladder tissue microorganisms may be causally linked to the development of BCa. Functional annotation analysis suggests a significant difference in microbiome function between MIBC and NMIBC tissues, but further studies are still needed to gain a deeper insight into the relationship between the microbial environment and the development of BCa.

## Data availability statement

The datasets presented in this study can be found in online repositories. The names of the repository/repositories and accession number(s) can be found below: Sequence Read Archive (SRA) of the National Center for Biotechnology Information (NCBI) under BioProject accession number PRJNA946904.

## Ethics statement

The studies involving human participants were reviewed and approved by Ethical Review Board of Tongji Hospital, Tongji Medical College, Huazhong University of Science and Technology (2021S055). The patients/participants provided their written informed consent to participate in this study.

## Author contributions

J-XS, Q-DX, X-YZ, ZL, and S-GW contributed to the conception and design of the study. J-XS and X-YZ conceived and drafted the manuscript and collected samples and clinical information from the patients. Q-DX and J-XS participated in the data process, analysis and interpretation. S-GW and ZL supervised the project and revised the manuscript. All authors contributed to the article and approved the submitted version.
