# Supplementary material for: The bladder microbiome of NMIBC and MIBC patients revealed by 2bRAD-M
Source: Front Cell Infect Microbiol. 2023 Jun 7;13:1182322. doi: 10.3389/fcimb.2023.1182322 (PMC10282653; doi:10.3389/fcimb.2023.1182322)
Supplement: Supplementary file 1 [file DataSheet_1.docx]

Supplementary Material

The bladder microbiome of NMIBC and MIBC patients revealed by 2bRAD-M

Jian-Xuan Sun^†^, Qi-Dong Xia^†^, Xing-Yu Zhong^†^, Zheng Liu^*^, Shao-Gang Wang^*^

**^†^**These authors contributed equally to this work and share first authorship

*** Correspondence:** Zheng Liu: lz2013tj@163.com; Shao-Gang Wang: sgwangtjm@163.com

# Supplementary Figures and Tables

## Supplementary Figures


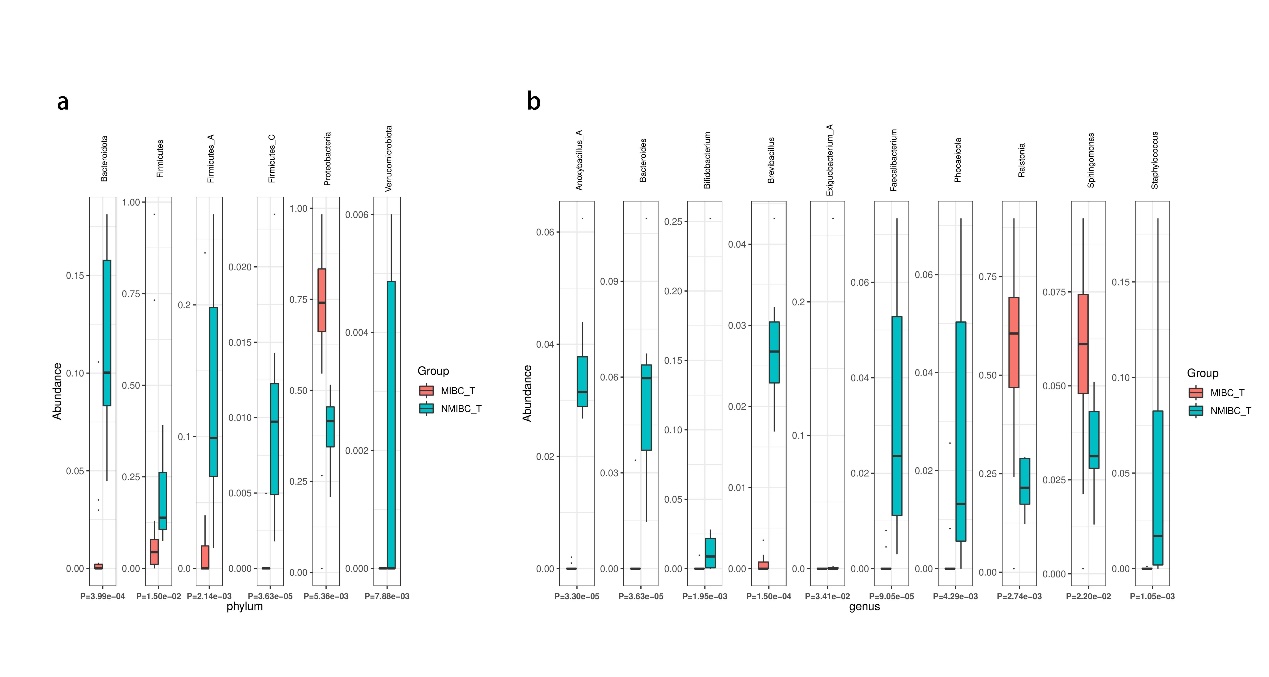


**Supplementary Figure 1.** Comparison of the differences in abundance of microbial taxa between MIBC and NMIBC groups. a) Six of the top ten phyla with significant differences in abundance. b) Top ten genera with significant differences.


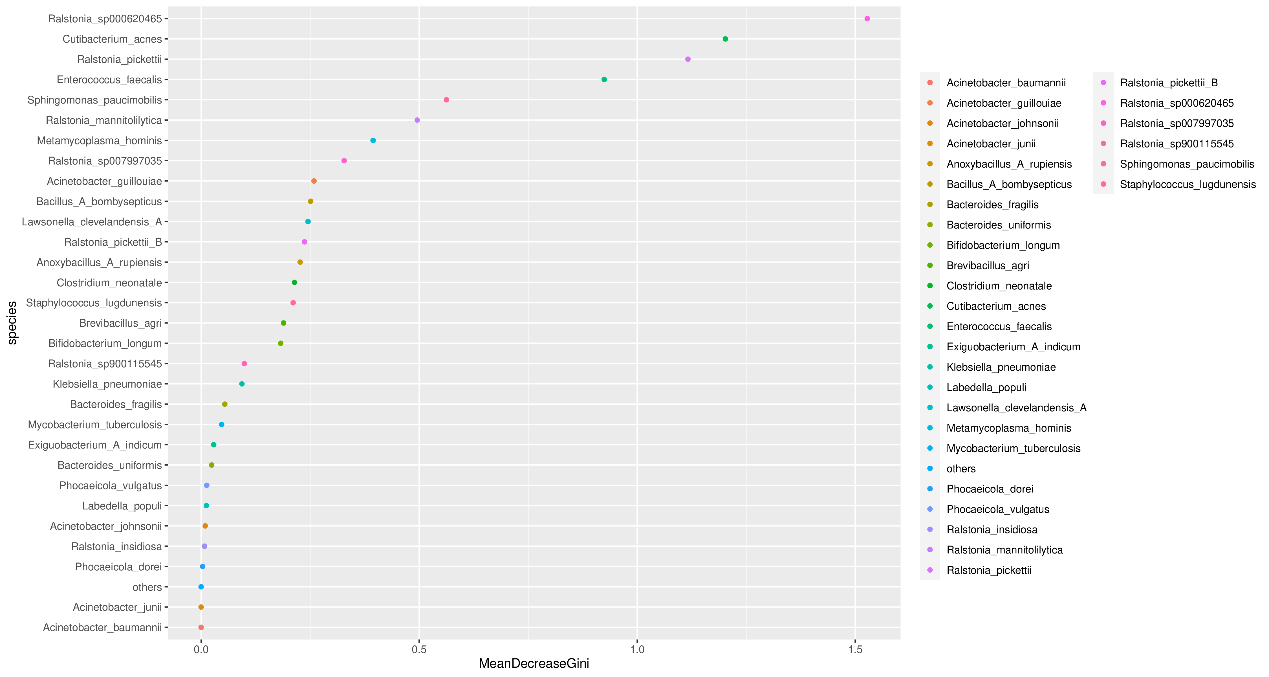


**Supplementary Figure 2.** Species importance point diagram. MeanDecreaseGini indicates the importance measure.

## Supplementary Tables

**Supplementary Table 1.** Details of the adapters and primer sequences used for 2bRAD-M library preparation.

| Adaptors and primers |
| --- |
| Adap-1 sense |
| ACACTCTTTCCCTACACGACGCTCTTCCGATCTNNN |
| Adap-1 antisense |
| AGATCGGAAGAGC(AminoC6) |
| Adap-2 sense |
| GTGACTGGAGTTCAGACGTGTGCTCTTCCGATCTNNN |
| Adap-2 antisense |
| AGATCGGAAGAGC(AminoC6) |
| Primer 1 |
| ACACTCTTTCCCTACACGACGCT |
| Primer 2 |
| GTGACTGGAGTTCAGACGTGTGCT |
| Primer 3 |
| AATGATACGGCGACCACCGAGATCTACACTCTTTCCCTACACGACGCT |
| Index primer |
| CAAGCAGAAGACGGCATACGAGATXXXXXXGTGACTGGAGTTCAGACGTGT |

**Supplementary Table 2.** Sequencing information summary: number of raw reads, enzyme reads, and clean reads and the percentage.

| Sample | Raw Reads | Enzyme Reads | Clean Reads (PassQc) | Percent |
| --- | --- | --- | --- | --- |
| MIBC_01T | 6708616 | 5641953 | 5329250 | 79.44% |
| MIBC_02T | 8475089 | 6914974 | 6555946 | 77.36% |
| MIBC_03T | 8746567 | 7553193 | 7136471 | 81.59% |
| MIBC_04T | 8118915 | 6722654 | 6362352 | 78.36% |
| MIBC_05T | 7104817 | 6171299 | 5820756 | 81.93% |
| MIBC_06T | 7862563 | 6663620 | 6280387 | 79.88% |
| MIBC_07T | 8339258 | 7610602 | 7184046 | 86.15% |
| MIBC_08T | 8470647 | 7459320 | 7052804 | 83.26% |
| MIBC_09T | 9149674 | 8238315 | 7799006 | 85.24% |
| MIBC_10T | 9404875 | 7731349 | 7323239 | 77.87% |
| MIBC_11T | 10847940 | 8755984 | 8293179 | 76.45% |
| MIBC_12T | 7026553 | 5360900 | 5072571 | 72.19% |
| MIBC_13T | 10279226 | 8736915 | 8289841 | 80.65% |
| MIBC_14T | 8185217 | 6560998 | 6197877 | 75.72% |
| MIBC_15T | 8658519 | 7204332 | 6800865 | 78.55% |
| NMIBC_1T | 9932430 | 8222319 | 7730484 | 77.83% |
| NMIBC_2T | 10301060 | 7777393 | 7327879 | 71.14% |
| NMIBC_3T | 11138884 | 8141849 | 7675222 | 68.90% |
| NMIBC_4T | 12902629 | 10407863 | 9814880 | 76.07% |
| NMIBC_5T | 11738232 | 9852221 | 9273097 | 79.00% |
| NMIBC_6T | 11889022 | 9464486 | 8923169 | 75.05% |
| NMIBC_7T | 9176240 | 7442958 | 7005043 | 76.34% |

**Supplementary Table 3.** List of the 5 species in the optimal marker set.

| 1 | Ralstonia_mannitolilytica |
| --- | --- |
| 2 | Ralstonia_pickettii_B |
| 3 | Bacteroides_uniformis |
| 4 | Lawsonella_clevelandensis_A |
| 5 | Klebsiella_pneumoniae |
